# Unique Case of a Hamartomatous Duodenal Polyp Associated With Intestinal Schistosomiasis

**DOI:** 10.14309/crj.0000000000000485

**Published:** 2021-01-29

**Authors:** Adalberto Gonzalez, Kapil Gupta, Sandra Rodriguez, Vaibhav Wadhwa, Pablo Bejarano, Roger Charles

**Affiliations:** 1 Department of Gastroenterology, Cleveland Clinic Florida, Weston, FL; 2 University of Miami/JFK Medical Center Palm Beach Consortium, Atlantis, FL; 3 Digestive Medicine Associates, Miami, FL; 4 Department of Pathology, Cleveland Clinic Florida, Weston, FL

## Abstract

Schistosomiasis is a trematode infection rarely diagnosed in the United States. Intestinal involvement is common with chronic infection and causes abdominal pain, changes in bowel habits, hematochezia, and polyp formation. Chronic, disseminated infection can affect the intestines causing the aforementioned symptoms, but reports of intestinal polyps are rare. Most cases are inflammatory fibrous polyps in the colon. There are very few cases reported in the literature of hamartomatous polyps arising in the small intestine. We present the rare case of a U.S.-born, 35-year-old woman diagnosed with a large duodenal hamartomatous polyp in the setting of intestinal schistosomiasis.

## INTRODUCTION

Schistosomiasis affects more than 200 million people worldwide but is rarely diagnosed in the United States.^1^ Gastrointestinal schistosomiasis can affect the liver or intestines.^2^ Intestinal schistosomiasis most commonly presents with colon polyps.^3,4^ To the best of our knowledge, there have been few reported cases of intestinal schistosomiasis in the United States.^8–10^ We know an international case of schistosomiasis that caused a hamartomatous colon polyp^5^ but no duodenal polyp related to schistosomiasis. We present a unique case of a 35-year-old woman and raised in the United States who developed a hamartomatous duodenal polyp that may have been secondary to intestinal schistosomiasis.

## CASE REPORT

A 35-year-old woman presented to the gastroenterology clinic for evaluation of dull abdominal pain. The abdominal pain started insidiously 2 years previous, was intermittent in nature, and located in the epigastric and left lower quadrant. The patient also complained of concomitant diarrhea, flatulence, and bloating. She denied melena, hematochezia, nausea, vomiting, fevers, chills, or weight loss. She had an upper endoscopy and colonoscopy 2 years earlier with no significant findings. She had a medical history notable for type 1 diabetes mellitus and a previous benign bladder tumor with unknown pathology that was surgically resected. She was born and raised in the United States and had lived in multiple cities within the United States. Her only travel outside of the United States had been during her childhood on vacation to the Bahamas, the Cayman Islands, and Mexico. She denied any wading or swimming in any freshwater source outside of the United States. She denied any cigarette smoking, alcohol use, or illicit drug use.

Her abdominal examination revealed no distention, no scars, normal bowel sounds, no tenderness, no organomegaly, and normal tympani. The rest of her physical examination was unremarkable. Her laboratory testing was within normal limits. Abdominal ultrasound was performed at a later time due to abdominal pain showed a 9-mm indeterminate hepatic lesion. Abdominal magnetic resonance with and without contrast showed multiple hyperenhancing liver lesions. The hepatic lesions were biopsied. It revealed non-necrotizing granulomas composed of multinucleated giant cells and cuffs of lymphocytes; moderate hepatic steatosis was present.

**Figure 1.**
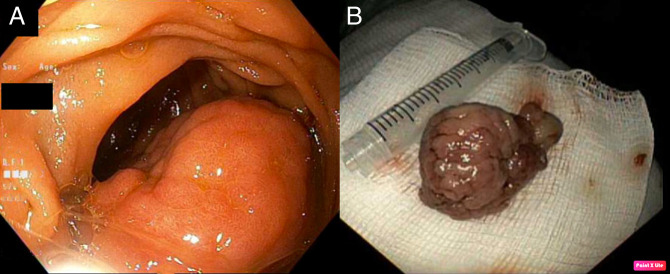
(A) The pedunculated polyp with no bleeding in the second part of the duodenum and (B) the resected polyp.

**Figure 2.**
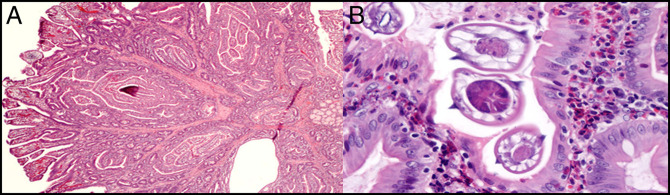
Pathology showing (A) adenomatous changes arising in a hamartomatous polyp (Peutz-Jeghers type) and (B) parasite organisms present in the crypts of the polyp consistent with schistosomiasis accompanied by tissue eosinophilia.

Upper endoscopy was performed to reveal a 40-mm pedunculated polyp with no bleeding in the second part of the duodenum; this was resected (Figure 1). The esophagus and stomach were normal. Colonoscopy revealed a 6-mm sessile polyp in the sigmoid colon; this was resected. Pathology from the duodenal polyp revealed adenomatous changes arising in a hamartomatous polyp (Peutz-Jeghers type); there was also the presence of parasite organisms present in the crypts of the polyp consistent with schistosomiasis with tissue eosinophilia (Figure 2). Pathology of the colon polyp revealed hyperplastic changes. The patient was diagnosed with a hamartomatous duodenal polyp due to *Schistosoma mansoni*. The patient was referred to infectious disease. She was prescribed praziquantel 20 mg/kg/dose 3 times per day for 1 day. The patient followed up with gastroenterology after treatment with praziquantel. Her diarrhea had resolved, but she continued to have intermittent left lower quadrant abdominal pain, attributed to irritable bowel syndrome. Repeat endoscopy showed no evidence of duodenal hamartomatous polyp.

## DISCUSSION

Although rare in the United States, schistosomiasis is one of the most widespread parasitic infections.^1,2,13^ Risk factors include exposure to contaminated freshwater in endemic areas. The patient in our case had a very remote travel history as an adolescent and did not have any exposure to any freshwater sources. The most important species infecting humans are *Schistosoma mansoni*, *S. japonicum*, *S. mekongi*, *S. haematobium*, and *S. intercalatum*.^1^ The *S. mansoni* life cycle involves an intermediate host (freshwater snail) and definitive host (human).^12^ They enter humans through the epidermis, and they travel using the lymphatic and circulatory system. Mature schistosomes are found mostly in the small inferior mesenteric blood vessels. They sexually reproduce, deposit the eggs to the gut and urinary bladder, and the eggs are excreted.

Intestinal schistosomiasis can also occur in the small or large intestine and may present with polyps, acute colitis, or cancer, depending on acuity of infection and species.^3,4^ The underlying mechanism starts with egg deposition in the loose superficial layers of submucosa and subsequently progresses into cell-mediated inflammatory response with granuloma formation to form polyps.^7,19^ Most polyps are inflammatory/fibrous, but hamartomatous, hyperplastic, and adenomatous polyps have been reported; they range in size from 2 to 20 mm and sessile, pedunculated, or cauliflower.^5,14,15,19^ The tumorigenic potential of polyps caused by intestinal schistosomiasis has been long been debated, but there may be a small risk of colorectal cancer,^2,17^ especially with *S. japonicum*.^6,18^ There are few cases of colorectal cancer that have been attributed to *S. japonicum*. Consequently, schistosomal infections have been linked to other cancers, such as the link between *S. haematobium* and bladder cancer.^18^ We did not find any link between intestinal schistosomiasis and bladder tumors in the literature search. Also, schistosoma-lodged eggs lead to granulomatous inflammation in the liver and healed by periportal fibrosis. Pathology shows granulomatous changes in the portal triad and may be consisted of lymphocytes, multinucleated giant cells, and eosinophils.^20^ We believe that explains the pathology of the liver lesions in our patient's case.

There have been a few reported cases of colon polyps in the absence of inflammation in endemic areas.^5–7^ However, intestinal involvement in schistosomiasis is usually confined to the ileum, colon,^16^ and rectum,^7,8^ but duodenal involvement can occasionally be present.^11,16^ Intestinal schistosomiasis of any kind in the United States is rare.^8–10^ We are aware of only 1 previous case of a duodenal polyp caused by intestinal schistosomiasis.^11^ The diagnosis of intestinal schistosomiasis requires egg identification from stool samples or endoscopy biopsy. Serology to detect antischistosomal antibodies in serum or urine can be helpful to rule out infection in the epidemic area, but its sensitivity and specificity can sometimes be challenging in the nonepidemic area.^7^ Treatment for schistosomiasis is relatively safe and effective, especially in the developed countries where the resistance to praziquantel has not been reported yet.^7^

## DISCLOSURES

Author contributions: A. Gonzalez, K. Gupta, and V. Wadhwa wrote the manuscript. S. Rodriguez and R. Charles edited the manuscript. P. Bejarano was the pathologist. K. Gupta is the article guarantor.

Financial disclosure: none to report.

Informed consent was obtained for this case report.
